# Analgesic effect of oral paracetamol 1000 mg/ibuprofen 400 mg, paracetamol 1000 mg/codeine 60 mg, paracetamol 1000 mg/ibuprofen 400 mg/codeine 60 mg, or placebo on acute postoperative pain: a single-dose, randomized, and double-blind study

**DOI:** 10.1007/s00228-023-03525-0

**Published:** 2023-06-22

**Authors:** Gaute Lyngstad, Per Skjelbred, David Michael Swanson, Lasse Ansgar Skoglund

**Affiliations:** 1 Section of Dental Pharmacology and Pharmacotherapy, Institute of Clinical Dentistry, Faculty of Dentistry, University of Oslo, Blindern, P. O. Box 1119, N-0317 Oslo, Norway; 2 Department of Maxillofacial Surgery, Oslo University Hospital, P. O. Box 4950 Nydalen, N-0424 Oslo, Norway; 3 Oslo Centre for Biostatistics and Epidemiology, Oslo University Hospital, Blindern, P.O. Box 1122, N-0317 Oslo, Norway

**Keywords:** Paracetamol, Ibuprofen, Codeine, Combination, Postoperative pain, Third molar

## Abstract

**Purpose:**

Combining analgesics with different mechanisms of action may increase the analgesic efficacy. The multidimensional pharmacodynamic profiles of ibuprofen 400 mg/paracetamol 1000 mg, ibuprofen 400 mg/paracetamol 1000 mg/codeine 60 mg, and paracetamol 1000 mg/codeine 60 mg and placebo were compared.

**Methods:**

A randomized, double-blind, placebo-controlled, parallel-group, single-centre, outpatient, and single-dose study used 200 patients of both sexes and homogenous ethnicity after third molar surgery (mean age 24 years, range 19–30 years). Primary outcome was sum pain intensity over 6 h (SPI). Secondary outcomes were time to analgesic onset, duration of analgesia, time to rescue drug intake, number of patients taking rescue drug, sum pain intensity difference (SPID), maximum pain intensity difference, time to maximum pain intensity difference, number needed to treat, prevent remedication and harm values, adverse effects, and patient-reported outcome measure (PROM).

**Results:**

Analgesia following ibuprofen and paracetamol combination with or without codeine was comparable. Both were better than paracetamol combined with codeine. Secondary variables supported this finding. Post hoc analysis of SPI and SPID revealed a sex/drug interaction trend in the codeine-containing groups where females experienced less analgesia. PROM showed a significant sex/drug interaction in the paracetamol and codeine group, but not in the other codeine-containing group. Especially females reported known and mild side effects in the codeine-containing groups.

**Conclusion:**

Codeine added to ibuprofen/paracetamol does not seem to add analgesia in a sex-mixed study population. Sex may be a confounding factor when testing weak opioid analgesics such as codeine. PROM seems to be more sensitive than traditional outcome measures.

**Trial registration:**

ClinicalTrials.gov June 2009 NCT00921700.

**Supplementary Information:**

The online version contains supplementary material available at 10.1007/s00228-023-03525-0.

## Introduction

Management of acute pain conditions after ambulatory surgery can be a predicament because it requires the selection of analgesic drugs, which balance between optimal pain relief and minimal adverse effects. The idea of combining analgesics with different mechanisms of action and adverse-effect profiles lead Cooper and Beaver to study the clinical efficacy of oral analgesics-antipyretics combined with weak opioids [1]. It is reasonable to hypothesize from their research that the combination of an “analgesic-antipyretic”, a NSAID, and a weak opioid with relatively similar pharmacokinetic characteristics would further enhance the analgesic efficacy due to the different modes of action of the drugs.

This hypothesis was tested by Breivik et al. [2] using various combinations of paracetamol (acetaminophen), diclofenac, and codeine on postsurgical dental impaction pain. Surprisingly, they found no significant difference in pain relief between the combination of diclofenac 100 mg, paracetamol 1000 mg and codeine 60 mg, and the combination of diclofenac 100 mg and paracetamol 1000 mg only, contrasting with the hypothesis. All drugs used in this trial [2], including the active morphine derivatives of codeine, had relatively similar pharmacokinetic properties [3–5]. Studies that are more recent also showed no significant difference in pain relief between paracetamol 1000 mg, ibuprofen 400 mg and codeine 60 mg versus paracetamol 1000 mg and ibuprofen 400 on postsurgical dental impaction pain or versus paracetamol 1000 mg on acute musculoskeletal pain following injuries [6, 7].

We found the lack of adjuvant weak opioid analgesia when added to a combination of an analgesic-antipyretic and a NSAID confounding relative to extensive clinical experience. Consequently, we carried out an internal validity study using an ethnically and demographic outpatient population as homogenously as possible in a controlled randomized multidimensional trial. We investigated the relative clinical analgesic value of codeine 60 mg added to paracetamol 1000 mg or paracetamol 1000/ibuprofen 400 mg versus ibuprofen 400 mg/paracetamol 1000 mg, or placebo on acute postoperative pain after third molar surgery.

## Methods

### Design and ethics

This was a prospective randomized, double blind, single dose, placebo-controlled, parallel group, single-centre, outpatient, and fixed-dose study. The design included a screening period (at least 14 days before surgery), surgical period, qualification period waiting for local anaesthesia to wear off (up to 6 h after surgery), and an observation period of 6 h. Patients provided written informed consent prior to any screening- and study-related procedures. The patients did not receive remunerations.

### Patients and exclusion/inclusion criteria

Eligible participants of both sexes aged between 18 and 30 years of Norwegian Caucasian origin referred to the Department of Maxillofacial Surgery, Oslo University Hospital, for surgical removal of impacted third molars were enrolled by LAS. They were included if they reached “moderate” on a 5-point Likert verbal rating scale (VRS) being “no, mild, moderate, severe, or very severe pain” verified by ≥ 4 on a horizontal 11-point numerical visual analogue rating scale (NRS) running from “no pain = 0” to “worst imaginable pain = 10” within the qualification period [8]. Exclusion criteria were ASA-classification > II; ongoing drug treatment except contraceptives; use of analgesics 3 days prior to the surgery; pregnancy or planned conception; or known contraindication to NSAIDs, paracetamol, or opioids.

### Randomization and blinding

Prior to the trial, LAS made a sequentially numbered medication allocation list using a computer-generated system (www.randomization.com, seed number 5431). The randomized list contained four treatment groups identified by the letters A to D, in blocks containing eight patients. A person not involved in the trial assigned each of the four trial drugs to the treatment groups according to the randomized list. Ibumetin^®^ (ibuprofen 200 mg, Nycomed Pharma, Norway), Pinex^®^ (paracetamol 500 mg, Actavis, Iceland), and Pinex Forte^®^ (paracetamol 500 mg/codeine phosphate hemihydrate 30 mg, Actavis, Iceland) were commercially purchased, processed, and identically blinded in unmarked gelatine capsules, by the Oslo University Hospital Pharmacy according to GMP standards. Each single-trial dose contained six capsules packed in sequentially numbered envelopes to be opened by the patients. A person not involved in the trial did the packaging according to the randomized list. Active drug doses were ibuprofen 400 mg/paracetamol 1000 mg, paracetamol 1000 mg/codeine 60 mg, and ibuprofen 400 mg/paracetamol 1000 mg/codeine 60 mg. Placebo were capsules filled with lactose. All persons involved in the trial were blind with respect to trial drug identity.

### Procedure

Two surgeons (GL and LAS) used a standardized method of surgery under local anaesthesia using lidocaine 20 mg/ml plus 12.5 µg/ml epinephrine (Xylocaine Dental Adrenalin^®^, Dentsply, Surrey, England) [9]. Standardized instruction on how to complete the clinical record forms (CRF) was given according to a pre-determined protocol. Patients reaching “moderate” pain on the VRS during the qualification period of 6 h self-administered the trial drug under supervision. The principal investigator (GL) was available in case of any serious adverse effects, or if any issues regarding the CRF emerged. Paracetamol 500 mg plus codeine 30 mg (Pinex Forte^®^) was available as rescue drugs. The patients visited the clinic 7 days after the day of surgery for postoperative control.

### Efficacy outcomes

Present pain intensity (PI) was rated on a horizontal NRS at 0 min (time of ingestion/baseline pain), 10, 20, 30, 40, 50, 60, 75, 90, and every 30 min up to 6 h post trial dose. The primary outcome measure was sum pain intensity (SPI) over 6 h after trial drug intake calculated by adding all the PI scores over 6 h. In the event of rescue drug intake, the PI score reverted to the baseline PI [10]. Secondary outcomes were time to analgesic onset defined as time between trial drug intake and first report of pain relief, duration of analgesia defined as the time between the first report of perceptible and meaningful pain relief and pain reappearing, time to rescue drug intake defined as the time from trial drug intake to intake of first rescue drug, number of patients taking rescue drug, number of rescue drug tablets, sum pain intensity difference (SPID) over 6 h, maximum pain intensity difference (MAXPID), and time to MAXPID. Pain intensity difference (PID) scores were calculated by subtracting the PI score at each time point from baseline PI, and SPID calculated equivalent to calculation of SPI. An overall assessment of the trial drugs made by the patients was used as a patient-reported outcome measure (PROM) using one each of the following alternatives: poor, fair, good, very good, or excellent, were used as a patient-reported outcome measure [11]. The point estimates number needed to treat (NNT) and number needed to prevent the use of rescue drug (NNTp), and number needed to harm (NNH) were calculated with placebo as comparator.

### Adverse effects

The patients were instructed to report any events considered an adverse effect (AE) related only to the trial drugs on the questionnaires, and by interview during the follow-up visit on the seventh postoperative day. AEs were labelled as none, mild, moderate, or severe, and the type of reported AE was recorded on the questionnaire.

### Sample size estimation

We used pooled standard deviation and effects measured in a previous trial using a similar patient population to determine sample size [12]. A sample size of 23 patients per active group would give 80% power to show a difference of at least 42% in SPI between paracetamol 1000 mg/codeine 60 mg and placebo with a two-tailed type 1 error rate of 0.05. Paracetamol 1000 mg with a SPI difference of 23%, the calculated sample size was 49. A theoretical sample size of 204 patients was necessary under the same condition to show a minimum SPI difference of 25% between paracetamol 1000 mg and paracetamol 1000 mg/codeine 60 mg. We judged such a sample size not to be relevant with respect to distinguishing clinically meaningful pain relief between active drugs within a homogenous patient population. We chose a sample size of 50 patients per treatment group.

### Statistical methods

All data were quality-checked after trial completion and locked for any corrections. The intention to treat (ITT) population was analysed blind. All clinical endpoints were first tested for an overall effect of the trial drug groups, and pairwise comparisons between groups were done if statistical significance was achieved with respect to overall group effect. Standard descriptive statistics such as mean, median, range, 95% confidence intervals (95% CI), and 1st and 3rd quartile (Q1, Q3) were used to describe the endpoints where appropriate for a useful comparison with previously published studies. The primary endpoint SPI and the secondary endpoints SPID, duration of analgesia, MAXPID, participants using rescue drug, and number of rescue drug tablets used were analysed with the various models of the *F*-test. Continuous demographic and surgical variables, baseline pain (NRS and VRS), and PROM were analysed with the Wilcoxon test. Baseline pain for females and males within each treatment group was analysed with the independent-samples Mann–Whitney *U* test. Sex and smoker distribution between groups, frequencies of AEs, sex distribution relating to AEs, sex/AE interaction, and sex distribution relating to age and BMI within groups were analysed with the Pearson chi-square test. For time-to-event outcomes (i.e. time to analgesic onset, time to MAXPID, and time to rescue drug), pairwise log-rank tests were used. Frequencies used for calculating NNT, NNTp, and NNH were analysed using an equal proportion *Z*-test. Responder NNT was defined as a SPI score ≤ 50% of SPI 0–360 using baseline PI [13]. Percentages of patients taking recue drug or no rescue drug were used for NNTp calculations. Post hoc analyses for sex/drug interactions were done for the primary variable SPI and the secondary variables SPID and PROM. SPI and SPID interaction with sex was tested with linear regression models, and PROM interaction with sex was tested with a proportional odds model. All tests were 2-sided and analysed with SPSS v. 24.0 and R version 3.5.2 [14, 15]. When multiple group analysis showed a statistical significant difference (*p* < 0.05) within an efficacy outcome, only significant *p* values for pair-wise differences between treatment groups are shown. Calculation of sample size was performed using PS: Power and Sample Size Calculations v. 2.1.30 [16] and a post hoc sample size analysis was performed with SPSS v. 28.0.

## Results

### Participant enrolment

Of 206 patients, 200 patients fulfilled the inclusion criteria between June 2009 and September 2010, completed the trial successfully, and were eligible for statistical analyses (Supplementary information 1). Six patients did not experience “moderate” pain. The study population consisted of 16.0% more females than males. There were no statistically significant differences between groups regarding patient and surgical characteristics (Supplementary information 2), or PI NRS between the groups when trial drugs were taken (Table 1). There was no statistically significant difference between females and males in each treatment group although in every group females scored somewhat numerically higher than males (Table 1). There were no significant differences between sexes with respect to BMI or age within each group (data not shown). Initial VRS was ≥ “moderate pain” for all patients.

**Table 1:** The primary outcome sum pain intensity over 6 h (SPI), SPI for each sex, baseline pain at drug intake, number needed to treat (NNT), number needed to prevent rescue drug intake (NNTp), and rescue drug details for each trial group are shown. Present pain was scored on numerical rating scales (0–10 NRS) ranging from

	Paracetamol 1000 mg Ibuprofen 400 mg	Paracetamol 1000 mg Codeine 60 mg	Paracetamol 1000 mg Ibuprofen 400 mg Codeine 60 mg	Placebo
	*n* = 50	*n* = 50	*n* = 50	*n* = 50
	F/M = 25/25	F/M = 28/22	F/M = 29/21	F/M = 26/24
*Pain at drug intake (NRS)*				
Median	6.0	5.0	5.5	5.0
(Q1, Q3)	(4.8, 7.0)	(4.0, 6.0)	(4.0, 6.3)	(4.0, 6.3)
Median (females)	6.0	5.5	6.0	6.0
(Q1, Q3)	(5.0, 7.0)	(4.3, 7.0)	(5.0, 7.0)	(4.8, 7.0)
Median (males)	6.0	5.0	5.0	5.0
(Q1, Q3)	(4.0, 7.0)	(4.0, 6.0)	(4.0, 6.0)	(4.0, 6.0)
Mean	6.0	5.5	5.5	5.5
(95% CI)	(5.6, 6.4)	(5.1, 5.9)	(5.1, 5.9)	(5.1, 5.9)
Mean (females)	6.2	5.8	5.8	5.7
(95% CI)	(5.6, 6.8)	(5.2, 6.4)	(5.3, 6.3)	(5.2, 6.3)
Mean (males)	5.7	5.2	5.2	5.3
(95% CI)	(5.2, 6.3)	(4.7, 5.7)	(4.6, 5.8)	(4.8, 5.7)
**Primary variable**				
*Sum pain intensity (SPI)*				
Median	44.5	62.5	39.5	89.0
(Q1, Q3)	(30.8, 56.5)	(40.0, 84.3)	(26.8, 56.3)	(75.0, 116.3)
Mean	47.6	65.2	43.5	94.3
(95% CI)	(42.2, 53.0)	(56.0, 74.4)	(37.7, 49.3)	(87.7, 100.9)
Median (females)	44.0	71.5	49.0	90.0
(Q1, Q3)	(33.5, 55.0)	(53.5, 92.5)	(33.0, 66.0)	(80.3, 119.3)
Mean (females)	46.2	73.3	50.7	95.7
(95% CI)	(39.5, 53.0)	(61.1, 85.5)	(42.7, 58.7)	(86.0, 105.4)
Median (males)	47.0	43.0	29.0	89.0
(Q1, Q3)	(28.0, 60.5)	(33.8, 69.3)	(26.0, 39.5)	(74.3, 115.8)
Mean (males)	48.9	54.8	33.6	92.8
(95% CI)	(40.1, 57.8)	(41.0, 68.6)	(26.6, 40.6)	(83.2, 102.3)
**Secondary variables**				
*NNT*	1.8	2.6	1.7	n/a
*NNTp*	1.5	5.0	1.7	n/a
*Rescue drug intake*				
Patients/no tablets	4/6	28/51	8/12	38/85

## Outcomes

### Primary endpoint

#### SPI

**Fig. 1:**
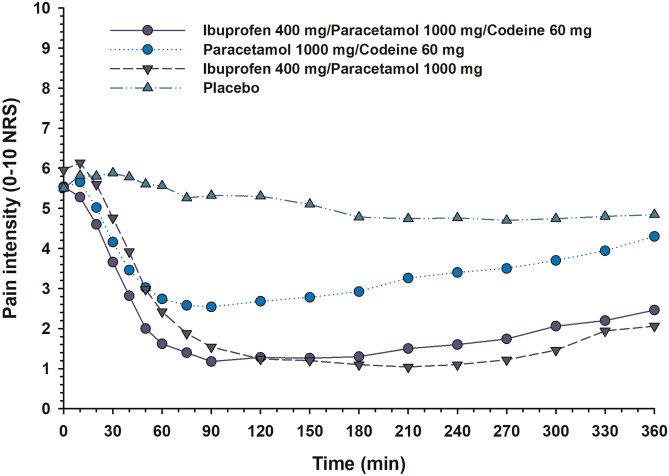
The graph shows the mean pain intensity scores (PI) on a numerical rating scale (0–10 NRS) after ibuprofen 400 mg/paracetamol 1000 mg, paracetamol 1000 mg/codeine 60 mg, ibuprofen 400 mg/paracetamol 1000 mg/codeine 60 mg, and placebo over the 6-h trial period. Missing data, due to intake of rescue drugs, are replaced with individual baseline pain values scored at trial drug intake

Ibuprofen 400 mg/paracetamol 1000 mg/codeine 60 mg (*p* < 0.0002) and ibuprofen 400 mg/paracetamol 1000 mg were not significantly different, but both had a significantly lower SPI than paracetamol 1000 mg/codeine 60 mg (*p* < 0.002) (Table 1). All active groups were superior to placebo (*p* < 0.0001). Figure 1 shows the mean group PIs over the 6-h observation period.

### Secondary endpoints

#### Time to analgesic onset

There were no significant differences between active treatment groups (Supplementary information 3), but all were superior to placebo (*p* < 0.0001).

### Duration of analgesia

Ibuprofen 400 mg/paracetamol 1000 mg had significantly longer analgesic duration than paracetamol 1000 mg/codeine 60 mg (*p* < 0.006), but not ibuprofen 400 mg/paracetamol 1000 mg/codeine 60 mg (Supplementary information 3), which tended to have a longer duration than paracetamol 1000 mg/codeine 60 mg (*p* = 0.080). All active groups were superior to placebo (*p* < 0.0001).

### Time to rescue drug

Ibuprofen 400 mg/paracetamol 1000 mg and ibuprofen 400 mg/paracetamol 1000 mg/codeine 60 mg showed a significantly a longer time than paracetamol 1000 mg/codeine 60 mg (both *p* < 0.0001), but they were not significantly different from each other (Supplementary information 3). All active groups were superior to placebo (*p* < 0.0005).

#### SPID

Ibuprofen 400 mg/paracetamol 1000 mg (*p* < 0.0001) and ibuprofen 400 mg/paracetamol 1000 mg/codeine 60 mg (*p* < 0.0002) had a significantly higher SPID than paracetamol 1000 mg/codeine 60 mg, but they were not significantly different from each other (Supplementary information 3). All active groups were superior to placebo (*p* < 0.0001). Supplementary information 4 shows the mean group PIDs over the 6-h observation period.

#### MAXPID

Ibuprofen 400 mg/paracetamol 1000 mg (*p* < 0.0001) and ibuprofen 400 mg/paracetamol 1000 mg/codeine 60 mg (*p* < 0.001) showed a higher MAXPID than paracetamol 1000 mg/codeine 60 mg, but they were not significantly different from each other (Supplementary information 3). All active groups were superior to placebo (*p* < 0.0001).

##### Time to MAXPID

Ibuprofen 400 mg/paracetamol 1000 mg/codeine 60 mg (*p* < 0.04) and paracetamol 1000 mg/codeine 60 mg (*p* < 0.0005) showed a much shorter time to MAXPID than ibuprofen 400 mg/paracetamol 1000 mg, which showed a much longer time than placebo (*p* < 0.05). There was no significant difference between ibuprofen 400 mg/paracetamol 1000 mg/codeine 60 mg and paracetamol 1000 mg/codeine 60 mg, or each of those versus placebo (Supplementary information 3).

#### PROM

Ibuprofen 400 mg/paracetamol 1000 mg and ibuprofen 400 mg/paracetamol 1000 mg/codeine 60 mg were rated better than paracetamol 1000 mg/codeine 60 mg (*p* < 0.008 and *p* < 0.0004, respectively), but they were not significantly different from each other (Table 2). All active groups were superior to placebo (*p* < 0.0001).

**Table 2:** Patient-reported outcome measure (PROM) made on a 5-point verbal rating scale (VRS) with the alternatives poor, fair, good, very good, and excellent, PROM for each sex, and within-group distribution of PROM scores are shown for each trial group

	Paracetamol 1000 mg Ibuprofen 400 mg	Paracetamol 1000 mg Codeine 60 mg	Paracetamol 1000 mg Ibuprofen 400 mg Codeine 60 mg	Placebo
	*n* = 50	*n* = 50	*n* = 50	*n* = 50
	F/M = 25/25	F/M = 28/22	F/M = 29/21	F/M = 26/24
*PROM (VRS)*				
Median	3.0	3.0	3.0	0.0
(Q1, Q3)	(3.0, 4.0)	(1.8, 3.0)	(3.0, 4.0)	(0.0, 1.0)
Mean	3.1	2.5	3.3	0.8
(95% CI)	(2.9, 3.3)	(2.2, 2.8)	(3.1, 3.5)	(0.5, 1.0)
Median (females)	3.0	2.0	3.0	0.0
(Q1, Q3)	(3.0, 4.0)	(1.0, 3.0)	(3.0, 4.0)	(0.0, 1.0)
Mean (females)	3.2	2.2	3.3	0.8
(95% CI)	(2.8, 3.6)	(1.8, 2.6)	(3.0, 3.5)	(0.3, 1.2)
Median (males)	3.0	3.0	3.0	0.5
(Q1, Q3)	(3.0, 4.0)	(2.0, 4.0)	(3.0, 4.0)	(0.0, 1.0)
Mean (males)	3.0	2.8	3.2	0.8
(95% CI)	(2.7, 3.2)	(2.3, 3.3)	(2.9, 3.6)	(0.4, 1.2)
*Distribution of PROM scores*				
*Ratio females/males and % of sum score*				
Excellent	11/4 30.0	3/7 20.0	11/7 36.0	1/0 2.0
Very good	10/16 52.0	9/9 36.0	15/13 56.0	1/2 6.0
Good	2/5 14.0	8/2 20.0	3/0 6.0	3/2 10.0
Fair	2/0 4.0	7/3 20.0	0/1 2.0	7/8 30.0
Poor	0/0 0.0	1/1 4.0	0/0 0.0	14/12 52.0
Sum	25/25 100.0	28/22 100.0	29/21 100.0	26/24 100.0

#### NNT

Ibuprofen 400 mg/paracetamol 1000 mg and ibuprofen 400 mg/paracetamol 1000 mg/codeine 60 mg had the lowest NNTs and were not significantly different from each other (Table 1), but different from paracetamol 1000 mg/codeine 60 mg (*p* < 0.0004 and *p* < 0.00007, respectively). All active groups were superior to placebo (*p* < 0.00001).

##### Number of patients taking rescue drug

Considerably fewer ibuprofen 400 mg/paracetamol 1000 mg and ibuprofen 400 mg/paracetamol 1000 mg/codeine 60 mg patients took rescue drugs than paracetamol 1000 mg/codeine 60 mg and placebo patients did (all *p* < 0.0005), but they were not significantly different from each other. Fewer paracetamol 1000 mg/codeine 60 mg patients took rescue drug than placebo patients did, but the groups were not significantly different (Table 1).

##### Number of tablets taken as rescue drug

The ibuprofen 400 mg/paracetamol 1000 mg and ibuprofen 400 mg/paracetamol 1000 mg/codeine 60 mg groups used considerably less rescue drugs than the paracetamol 1000 mg/codeine 60 mg or the placebo group (all *p* < 0.0005), but they were not significantly different. Less rescue drug was used in the paracetamol 1000 mg/codeine 60 mg group compared to the placebo group, but the groups were not significantly different (Table 1).

##### NNTp

Ibuprofen 400 mg/paracetamol 1000 mg and ibuprofen 400 mg/paracetamol 1000 mg/codeine 60 mg had the lowest NNTp values and were not significantly different from each other (Table 1), but different from paracetamol 1000 mg/codeine 60 mg (both *p* < 0.00001). All active drugs were different from placebo (*p* < 0.00003).

##### Adverse effects

Females and males reported relatively few AEs (Table 3). They were mild to moderate, and all patients recovered within a short time. There were no significant differences between the groups. More females reported adverse effects than males (*p* < 0.005).

**Table 3:** Number needed to harm (NNH), total reported adverse effects broken down by sex, and types of reported adverse effects are shown for each trial group

	Acetaminophen 1000 mg Ibuprofen 400 mg		Acetaminophen 1000 mg Codeine 60 mg		Acetaminophen 1000 mg Ibuprofen 400 mg Codeine 60 mg		Placebo	
	*n* = 50		*n* = 50		*n* = 50		*n* = 50	
*NNH*	50.0		16.7		12.5		n/a	
*Adverse effects*								
No. reported	6		8		9		5	
Female/male	(5/1)		(6/2)		(8/1)		(2/3)	
Reported adverse effects	Female	Male	Female	Male	Female	Male	Female	Male
	2 fatigue 1 stomach pain 1 drowsiness 1 nausea and fatigue	1 fatigue	2 nausea 1 dizziness 1 nausea and stomach pain 1 nausea and dizziness 1 nausea and emesis	1 fatigue 1 stomach pain	3 dizziness and fatigue 1 fatigue 1 dizziness 1 nausea and dizziness 1 nausea, emesis, dizziness and chills 1 feeling intoxicated	1 dizziness	1 nausea 1 nausea, dizziness and headache	1 stomach pain 1 drowsiness 1 fever

#### NNH

The NNH value for ibuprofen 400 mg/paracetamol 1000 mg was higher than the other active groups (Table 3), but no significant difference was found between the active groups. Only paracetamol 1000 mg/codeine 60 mg was significantly different from placebo (*p* < 0.03).

#### Post hoc analyses for sex/drug interaction

There were no significant statistical differences between females and males in the active treatment groups for the primary variable SPI. However, an interesting numerical difference supported by a statistical trend was found between sexes in the codeine containing paracetamol 1000 mg/codeine 60 mg (*p* = 0.10) and ibuprofen 400 mg/paracetamol 1000 mg/codeine 60 mg (*p* = 0.14) groups where females score numerically higher SPI than males (Table 1). The difference between females and males in PI over the 6-h observation period after paracetamol 1000 mg/codeine 60 mg contrasting with no difference after placebo is shown as a graph in supplementary information 5. A power calculation using the mean and SD values from females and males in the paracetamol 1000 mg/codeine 60 mg group and a ratio of 1 shows that a sample size of more than 46 patients would be needed to achieve a statistical significant difference.

Supplementary information 6 shows the PI after ibuprofen 400 mg/paracetamol 1000 mg and ibuprofen 400 mg/paracetamol 1000 mg/codeine 60 mg. It is interesting to note that the PI graphs from females and males in both treatment groups are convergent until a time between 150 and 180 min. From that time females and males diverge in both treatment groups where especially males have less PI than females in the ibuprofen 400 mg/paracetamol 1000 mg/codeine 60 mg group. PID graphs support these observations (data not shown).

There was a similar pattern regarding SPID where males had a numerically higher score than females in the paracetamol 1000 mg/codeine 60 mg (*p* = 0.16) and ibuprofen 400 mg/paracetamol 1000 mg/codeine 60 mg (*p* = 0.1) groups, although the difference was not statistically significant (Supplementary information 3). A statistically significant sex/drug interaction was found in the PROM assessments. PROM score by females were lower than males in the paracetamol 1000 mg/codeine 60 mg (*p* < 0.007) group. Females had a numerically higher PROM score than males in the ibuprofen 400 mg/paracetamol 1000 mg group which tended to be statistically significant (*p* = 0.07). Interestingly, the PROM scores were almost equal regarding females and males in the ibuprofen 400 mg/paracetamol 1000 mg/codeine 60 mg group (Table 2).

## Discussion

Few clinical trials have been published at present time studying the effect of multimodal postoperative analgesia using a paracetamol/NSAID/codeine combination on acute post-operative/-traumatic pain [2, 6, 7]. Most pharmacodynamic variables for the mixed-sex populations in our study showed paracetamol 1000 mg/codeine 60 mg to be inferior to ibuprofen 400 mg/paracetamol 1000 mg and ibuprofen 400 mg/paracetamol 1000 mg/codeine 60 mg. Ibuprofen 400 mg/paracetamol 1000 mg and ibuprofen 400 mg/paracetamol 1000 mg/codeine 60 mg both showed better MAXPID than paracetamol 1000 mg/codeine 60 mg. Ibuprofen 400 mg/paracetamol 1000 mg had a significantly longer analgesic duration than paracetamol 1000 mg/codeine 60 mg, while analgesia following ibuprofen 400 mg/paracetamol 1000 mg/codeine 60 mg tended to last longer than paracetamol 1000 mg/codeine 60 mg. Both codeine-containing groups had a shorter time to MAXPID than ibuprofen 400 mg/paracetamol 1000 mg.

Pharmacokinetic interaction has been suggested as an explanation for the prolonged analgesic efficacy for paracetamol/NSAID combinations [2], but therapeutic fixed-dose ibuprofen/paracetamol combinations does not change the pharmacokinetic parameters of either drug [17] nor does the addition of codeine [18, 19]. Available pharmacokinetic studies do not seem to explain the prolonged analgesia of the ibuprofen/paracetamol combinations. Time-sensitive pharmacodynamic interactions between the drugs possibly due to different mechanisms and compartments of action may explain our findings.

The SPI supported by the SPID results in our study showed that ibuprofen 400 mg/paracetamol 1000 mg/codeine 60 mg did not provide general mean analgesic superiority compared to ibuprofen 400 mg/paracetamol 1000 mg in our mixed-sex study population suggesting that the ibuprofen/paracetamol component, and not the codeine component, provides the major analgesic effect. The results from our study and previous studies [2, 6, 7] may seem difficult to interpret since meta-analyses of acute pain studies show that paracetamol added to a NSAID increases analgesic efficacy compared to paracetamol alone, and codeine added to paracetamol, or ibuprofen shows a small, but proven analgesic advantage [20–22].

We were surprised at the apparently lack of analgesic efficacy of codeine in our ethnically homogenous study. Female participation in clinical trials is advocated [23]. Accordingly, we included females in our study, but females are more inclined to volunteer in clinical intervention studies than males [24]. This phenomenon makes it more difficult to create sex-balanced trial groups studying drug effects. Since our study comprised 16.0% more females than males, we choose to make a post hoc analysis of the primary variable for sex/drug interactions within each active treatment group. We were surprised to find that females had higher SPI scores in both codeine-containing groups than males contrasting with the ibuprofen/paracetamol and the placebo group. The same pattern of a difference was found in the SPID scores, although the sex differences found with SPI and SPID were not statistically significant.

Our sex/drug interaction findings relating to outcome measures regarding drug efficacy are interesting since the patient populations used in the previous trials using triple drug combinations consisted of multi-ethnic patient populations [6, 7] and significant female/male imbalances pertinent to relevant test groups [2, 6, 7]. Using non-homogenous patient populations and not considering a possible sex/drug interaction may have contributed to underestimation of the analgesic efficacy of codeine in those studies.

Sex- and gender-based pharmacological responses to drugs are known, but clinical drug trials comparing analgesic effects of different opioid analgesics in females and males are sparse. Some trials have shown that females experience more analgesia than males when treated with opioid agonists such as pentazocine and butorphanol both more selective for the kappa-receptor than the mu-receptor in acute postoperative pain [25, 26]. Another study investigated the analgesic effect of the prototype mu-receptor agonist morphine titrated intravenously in comparative doses to male and female postoperative patients according to body weight. This large-scale prospective study found that females experienced more severe pain and required a greater dose of morphine than males, although this sex difference seemed to decrease in elderly patients [27].

Newer studies with positron emission tomography of the human brain using highly selective radiotracers for kappa-receptors and mu-receptors show that males have higher kappa-receptor availability than females, but females have higher mu-receptor availability than males [28, 29]. It is interesting that the sex difference in mu-receptor availability seems to decrease with increasing age [29].

Metabolic conversion to morphine, and possibly the 6-glucuronide metabolite of morphine, is generally suggested to explain the analgesic efficacy of codeine, while others claim an analgesic contribution from codeine or its 6-glucuronide metabolite based on animal and human experiments [30, 31]. The selectivity for mu-receptors is much greater for morphine and its 6-glucuronide metabolite than codeine and its 6-glucuroide metabolite, while they all have much lower affinity for kappa-receptors than mu-receptors in animal brain tissue experiments [32]. These findings should create conditions for a good analgesic effect of codeine in female patients, but a prominent analgesic effect of codeine in females seems to be restricted in our study. It might be argued that CYPD2D6 polymorphism among the trialists causing little or no metabolism of codeine could have affected our results. In our study, there were no significant differences between sexes within the groups regarding age and BMI. The prevalence of poor metabolizers is reported to be 5–11% in a Caucasian population [33]. It seems less likely that this fact by itself should have a significant impact on our results considering the number of patients in each group. It should be noted that we are at present not aware of any studies describing any sex-dependent prevalence of CYP2D6 polymorphism in Caucasians. We propose that codeine doses in commonly available OTC formulations are too small regarding optimal analgesia in females if we exclude the possibility of increased opioid induced adverse effects.

Patients assess their subjective present pain intensity on linear scales such as the 0–10 point NRS used in our study. PID conversion is traditionally used as a measure for drug efficacy (i.e. pain reduction capability) to correct for individual baseline variability, but pain does not increase linearly [34]. Therefore, numerically calculated differences between pain scores may not accurately describe the individual analgesic efficacy of a drug. Changes of PI may be a more accurate descriptor of analgesic relief than changes in PID, because the latter require recall of the initial pain, and the cognitive ability to assess the degree to which current pain intensity differs from starting pain intensity [35]. The choice of SPI as our primary variable may explain the slight difference between the SPI and SPID results regarding sex/drug interaction analyses in our trial [36].

Adverse effects in our study were relatively few representing known issues. The NNH value from ibuprofen 400 mg/paracetamol 1000 mg was higher than, but not significantly different from the NNH values from the other active treatment groups. Only paracetamol 1000 mg/codeine 60 mg was significantly different from placebo. All episodes resolved within a short time, but there was a significant majority of complaints from female participants. The study showed a slight majority of adverse effects related to the use of the codeine-containing drugs used by females, something that is well known when using opioid analgesics [37].

It is noteworthy that the PROM appraisal of ibuprofen 400 mg/paracetamol 1000 mg/codeine 60 mg was roughly the same for both sexes contrasting with significant less female preference for paracetamol 1000 mg/codeine 60 mg and a strong female preference trend for ibuprofen 400 mg/paracetamol 1000 mg. This finding may seem paradoxical if the PROM scores are compared with the sex-based incidence (F/M) of adverse effects reported for the paracetamol 1000 mg/codeine 60 mg group (6/2) and the ibuprofen 400 mg/paracetamol 1000 mg/codeine 60 mg (8/1) groups, as they were nearly the same. Our results support PROM as a global assessment of analgesic drug effect during a defined postoperative period and encompass more quality of life parameters than solely a change in pain experience as shown with SPI and SPID scores.

We used the well-characterized third molar surgery model [38] conducted as a single-centre study with a very homogenous population, and defined entry pain. Weaknesses in our study were limitation to younger patients in need of third molar removals, homogenous Caucasian ethnicity, and the majority of female participants in the trial groups that may restrict generalization of the results. The sex distribution in our study may have reduced the inherent analgesic efficacy of codeine relative to the ibuprofen/paracetamol combinations.

Our study showed very good generalized analgesic outcomes of the combination of paracetamol and ibuprofen with or without codeine versus codeine added to paracetamol alone in a study with a sex-mixed trial population. NNT found in our study for paracetamol 1000 mg/codeine 60 mg was 2.6 compared with current published data (2.2, 95% CI 1.8–2.9) [21] indicating an overestimation of its analgesic efficacy in mixed-pain meta-analyses. NNT for ibuprofen 400 mg/paracetamol 1000 mg in our study was 1.8 compared with current published data (1.8, 95% CI 1.6, 2.2) from dental postsurgical pain studies [39]. NNT for ibuprofen 400 mg/paracetamol 1000 mg/codeine 60 mg was 1.7 in our study. No published NNT value compared with placebo is currently found for this combination.

The paucity of codeine as an analgesic enhancer in our trial for female patients may explain the difficulty in finding a clinically relevant adjuvant analgesic effect of codeine using generalized outcome data. The authors are aware of the overuse of weak opioids in the clinic with a significant potential for drug dependence, but there is a need for analgesics with more analgesic potency than NSAIDs and paracetamol or their combination. Correct clinical cost/benefit assessments including knowledge of analgesic sex-drug interactions are necessary to achieve this purpose. The possibility of an analgesic sex/drug interaction as shown in our study provides an argument for more studies into personalized sex-based differences in OTC analgesics.

### Supplementary Information

Below is the link to the electronic supplementary material.

Supplementary file1 (PDF 46 KB)

Supplementary file2 (PDF 334 KB)

Supplementary file3 (PDF 104 KB)

Supplementary file4 (PDF 21 KB)

Supplementary file5 (PDF 68 KB)

Supplementary file6 (PDF 75 KB)

## Data Availability

The data that support the findings of this study are available on reasonable request from the corresponding author.
