# Correction to: Analgesic effect of oral paracetamol 1000 mg/ibuprofen 400 mg, paracetamol 1000 mg/codeine 60 mg, paracetamol 1000 mg/ibuprofen 400 mg/codeine 60 mg, or placebo on acute postoperative pain: a single‑dose, randomized, and double‑blind study

**DOI:** 10.1007/s00228-024-03688-4

**Published:** 2024-04-24

**Authors:** Gaute Lyngstad, Per Skjelbred, David Michael Swanson, Lasse Ansgar Skoglund

**Affiliations:** 1 Section of Dental Pharmacology and Pharmacotherapy, Institute of Clinical Dentistry, Faculty of Dentistry, University of Oslo, Blindern, P. O. Box 1119, N‑0317 Oslo, Norway; 2 Department of Maxillofacial Surgery, Oslo University Hospital, Nydalen, P. O. Box 4950, N‑0424 Oslo, Norway; 3 Oslo Centre for Biostatistics and Epidemiology, Oslo University Hospital, Blindern, P.O. Box 1122, N‑0317 Oslo, Norway

**Correction to: European Journal of Clinical Pharmacology (2023) 79:1131–1141** 10.1007/s00228-023-03525-0

The image of Supplementary material 5 and 6 are the same. The correct image of Supplementary material 6 should be:



The Original article has been corrected.

