# Supplementary figures and images for: Analgesic effect of oral paracetamol 1000 mg/ibuprofen 400 mg, paracetamol 1000 mg/codeine 60 mg, paracetamol 1000 mg/ibuprofen 400 mg/codeine 60 mg, or placebo on acute postoperative pain: a single-dose, randomized, and double-blind study
Source: Eur J Clin Pharmacol. 2023 Jun 22;79(8):1131–41. doi: 10.1007/s00228-023-03525-0 (PMC10361915; doi:10.1007/s00228-023-03525-0)

Enrollment

Allocation

Follow-Up

Analysis

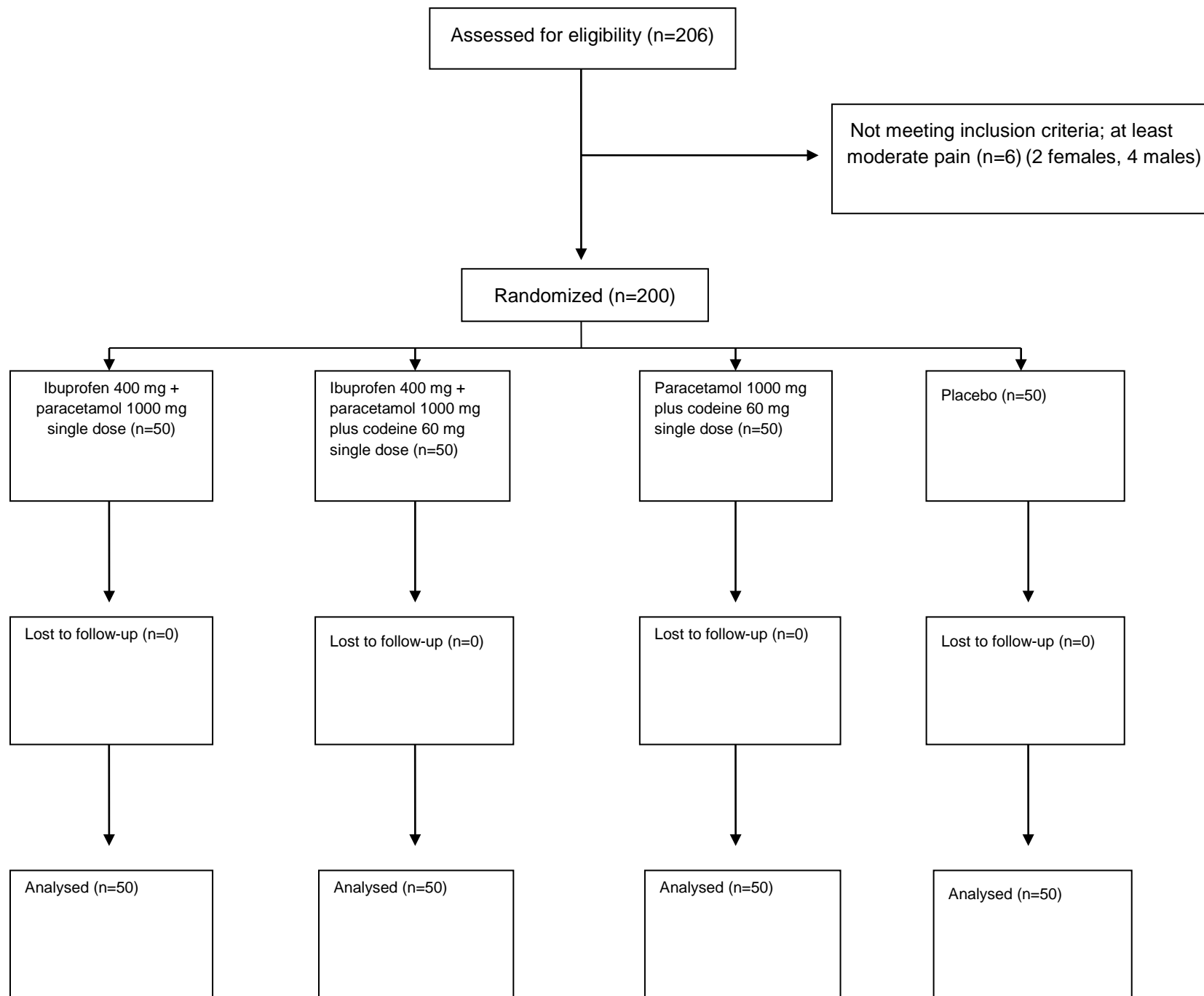

Supplement: Supplementary file 1 — Supplementary file1 (PDF 46 KB) [file 228_2023_3525_MOESM1_ESM.pdf]

Pain intensity difference score

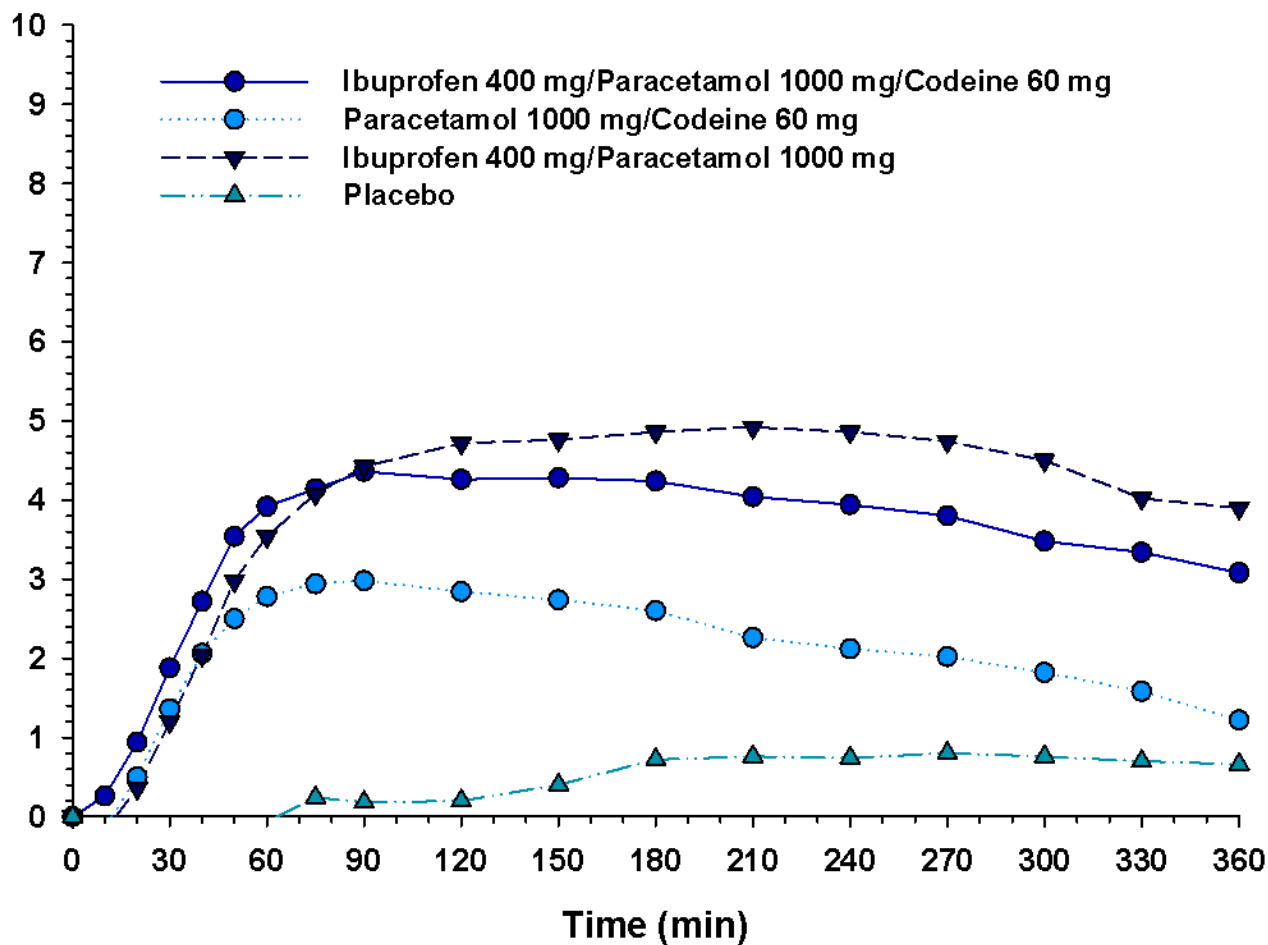

Supplement: Supplementary file 4 — Supplementary file4 (PDF 21 KB) [file 228_2023_3525_MOESM4_ESM.pdf]

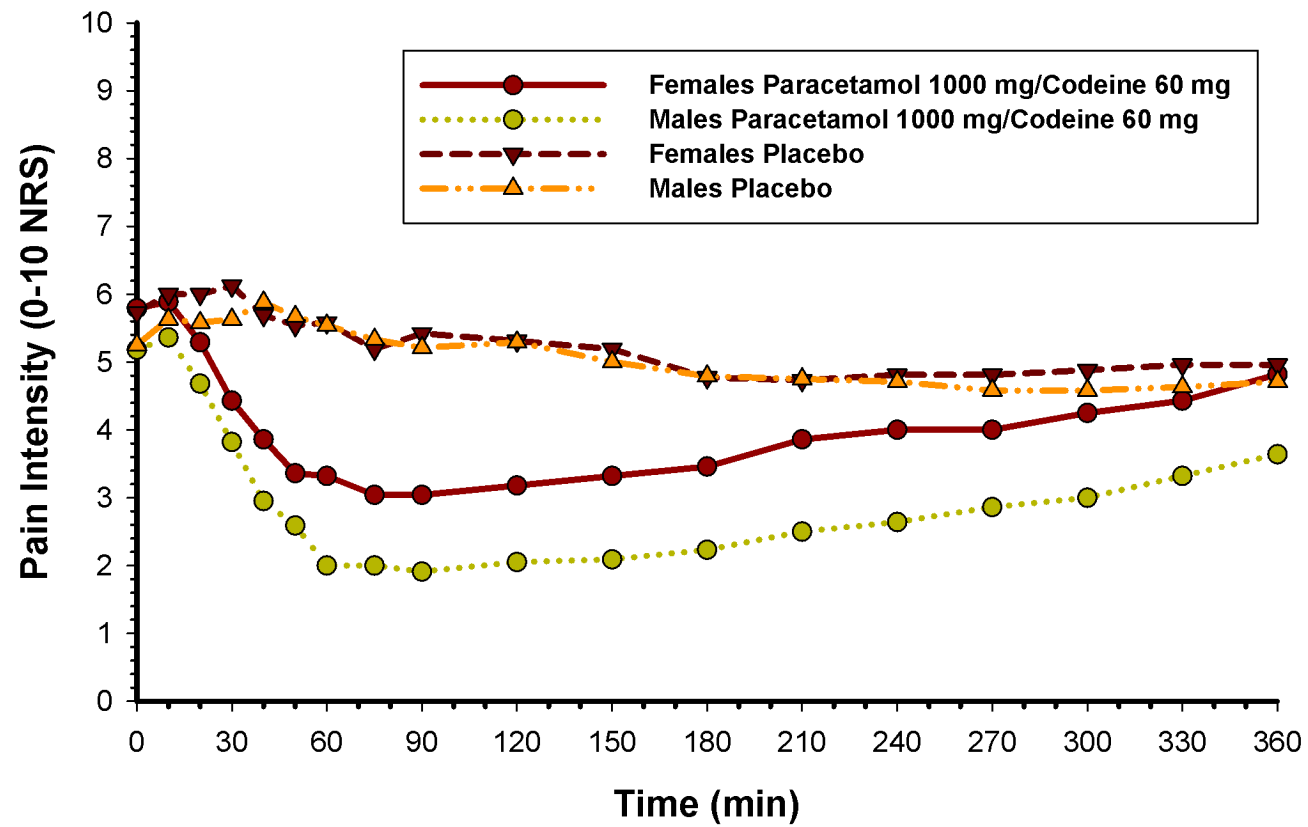

Supplement: Supplementary file 5 — Supplementary file5 (PDF 68 KB) [file 228_2023_3525_MOESM5_ESM.pdf]

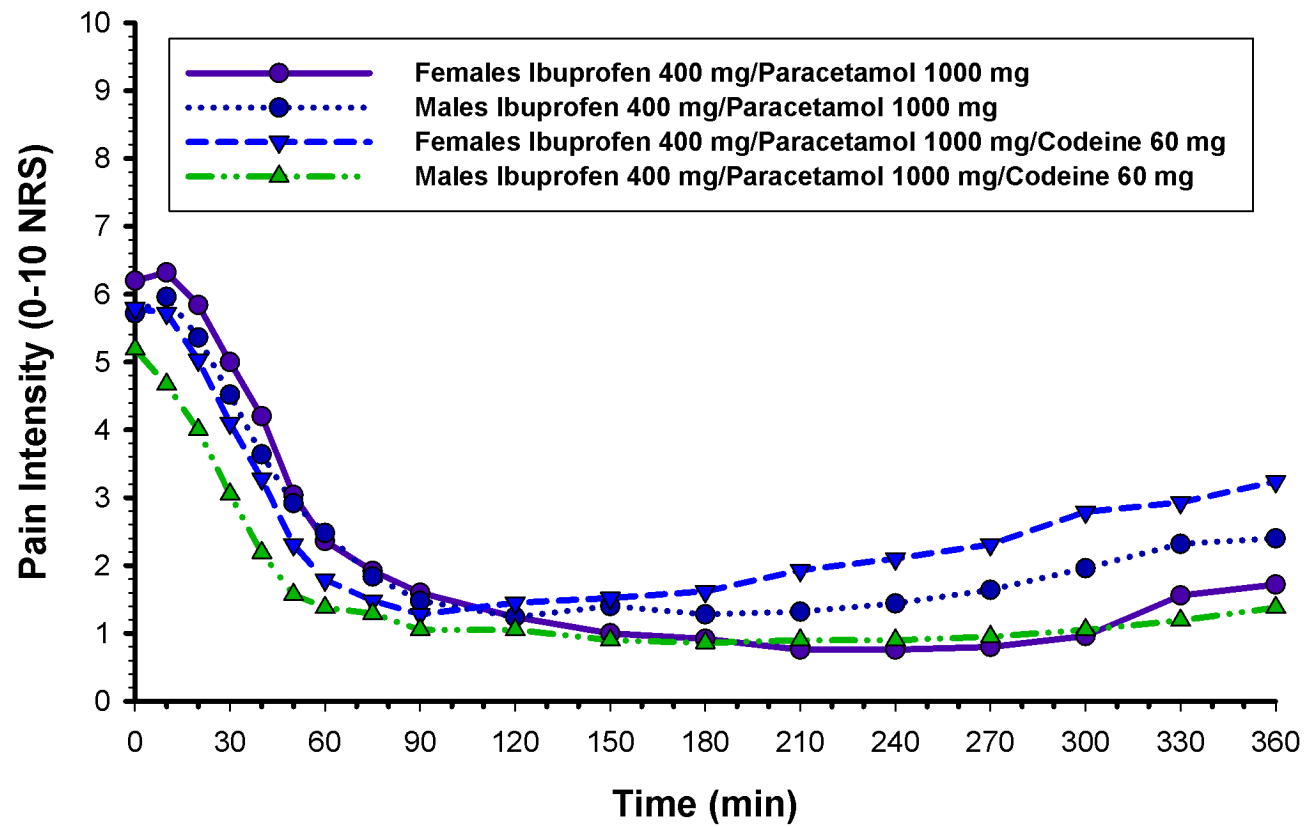

Supplement: Supplementary file 6 — Supplementary file6 (PDF 75 KB) [file 228_2023_3525_MOESM6_ESM.pdf]
