# Supplementary material for: Analgesic effect of oral paracetamol 1000 mg/ibuprofen 400 mg, paracetamol 1000 mg/codeine 60 mg, paracetamol 1000 mg/ibuprofen 400 mg/codeine 60 mg, or placebo on acute postoperative pain: a single-dose, randomized, and double-blind study
Source: Eur J Clin Pharmacol. 2023 Jun 22;79(8):1131–41. doi: 10.1007/s00228-023-03525-0 (PMC10361915; doi:10.1007/s00228-023-03525-0)
Supplement: Supplementary file 2 — Supplementary file2 (PDF 334 KB) [file 228_2023_3525_MOESM2_ESM.pdf]

	All patients	Paracetamol 1000 mg Ibuprofen 400 mg	Paracetamol 1000 mg Codeine 60 mg	Paracetamol 1000 mg Ibuprofen 400 mg Codeine 60 mg	Placebo
	n=200	n=50	n=50	n=50	n=50
Age (years)					
Median	24	24	24	25	25
(Q1, Q3)	(22, 26)	(22, 26)	(22, 26)	(22, 26)	(22, 26)
Mean	24	24	24	24	25
(Range)	(19-30)	(19-30)	(19-30)	(19-30)	(19-30)
BMI					
Median	22.6	23.0	22.8	22.0	23.0
(Q1, Q3)	(21.3, 24.2)	(21.5, 24.3)	(21.3, 24.9)	(21.5, 23.1)	(21.2, 24.9)
Mean	22.8	22.9	23.0	22.3	23.0
(95% CI)	(22.5, 23.1)	(22.2, 23.5)	(22.3, 23.7)	(21.9, 22.8)	(22.2, 23.8)
Females/Males (%)	54/46	50/50	56/44	58/42	52/48
Smokers (%)	17	22	16	18	12
Volume local anaesthesia (ml)					
Median	3.6	4.1	3.6	3.8	3.6
(Q1, Q3)	(3.6, 4.5)	(3.6, 5.4)	(3.6, 4.5)	(3.6, 4.6)	(3.2, 4.5)
Mean	4.2	4.4	4.1	4.1	4.1
(95 % CI)	(4.0, 4.3)	(4.1, 4.7)	(3.8, 4.4)	(3.8, 4.4)	(3.8, 4.5)
Duration of surgery (min)					
Median	17	20	15	17	17
(Q1, Q3)	(14, 25)	(15, 30)	(15, 23)	(12, 22)	(12, 21)
Mean	20	22	19	19	19
(95 % CI)	(18, 21)	(20, 24)	(16, 22)	(17, 21)	(16, 22)
