# Supplementary material for: Analgesic effect of oral paracetamol 1000 mg/ibuprofen 400 mg, paracetamol 1000 mg/codeine 60 mg, paracetamol 1000 mg/ibuprofen 400 mg/codeine 60 mg, or placebo on acute postoperative pain: a single-dose, randomized, and double-blind study
Source: Eur J Clin Pharmacol. 2023 Jun 22;79(8):1131–41. doi: 10.1007/s00228-023-03525-0 (PMC10361915; doi:10.1007/s00228-023-03525-0)
Supplement: Supplementary file 3 — Supplementary file3 (PDF 104 KB) [file 228_2023_3525_MOESM3_ESM.pdf]

Secondary variables	Paracetamol 1000 mg<br>Ibuprofen 400 mg<br><br>n=50<br>F/M=25/25	Paracetamol 1000 mg<br>Codeine 60 mg<br><br>n=50<br>F/M=28/22	Paracetamol 1000 mg<br>Ibuprofen 400 mg<br>Codeine 60 mg<br>n=50<br>F/M=29/21	Placebo<br><br><br>n=50<br>F/M=26/24
Time to analgesic onset (min)				
Median	30	27	30	365
(25, 75 quart)	(20, 39)	(20, 36)	(20, 39)	(54, 365)
Mean	31	51	30	255
(95 % CI)	(27, 35)	(27, 75)	(26, 34)	(212, 298)
Duration of analgesia (min)				
Median	340	212	325	0
(25, 75 quart)	(308, 409)	(139, 311)	(257, 370)	(0, 146)
Mean	381	215	341	75
(95 % CI)	(331, 431)	(183, 248)	(291, 391)	(40, 109)
Time to rescue drug (min)				
Median	365	332	365	100
(25, 75 quart)	(365, 365)	(206, 365)	(365, 365)	(62, 331)
Mean	356	279	339	166
(95% CI)	(346, 367)	(249, 308)	(320, 358)	(129, 202)
Sum pain intensity difference (SPID)				
Median	60.5	33.5	52.0	3.0
(Q1, Q3)	(44.0, 72.3)	(12.0, 53.5)	(37.8, 70.0)	(-5.5, 11.0)
Mean	59.7	34.2	56.2	4.7
(95% CI)	(53.5, 66.0)	(26.6, 41.8)	(48.3, 64.2)	(0.2, 9.2)
Median (Females)	65.0	22.5	50.0	4.0
(Q1, Q3)	(51.0, 81.5)	(10.0, 47.8)	(31.5, 66.0)	(-2.3, 15.3)
Mean (Females)	65.4	30.8	53.6	7.8
(95% CI)	(56.5, 74.3)	(20.2, 41.4)	(41.8, 65.4)	(0.3, 14.6)

Median (Males)	49.0	38.5	60.0	0.0
(Q1, Q3)	(43.0, 71.0)	(17.3, 65.0)	(41.0, 79.5)	(-8.0, 9.5)
Mean (Males)	54.0	38.5	59.9	1.8
(95% CI)	(45.2, 62.9)	(27.0, 49.9)	(49.3, 70.4)	(-3.8, 7.3)
Time to MaxPID (min)				
Median	120	60	60	30
(Q1, Q3)	(75.0, 150)	(30, 90)	(50,120)	(20,128)
Mean	118	75	89	81
(95 % CI)	(104, 132)	(55, 96)	(70, 107)	(54, 108)
MaxPID				
Median	5.0	4.0	5.0	1.0
(Q1, Q3)	(4.0, 6.3)	(2.8, 5.0)	(4.0, 6.0)	(-2.0, 2.0)
Mean	5.2	3.3	4.8	0.4
(95 % CI)	(4.8, 5.6)	(2.7, 4.0)	(4.3, 5.3)	(-0.3, 1.2)
